# The effectiveness of Parenthood-Centred Psychotherapy on perinatal maternal depressive-anxiety symptoms and child development in a disadvantaged population

**DOI:** 10.3389/fpsyg.2026.1758157

**Published:** 2026-03-25

**Authors:** Marta Gomà, Eulàlia Arias-Pujol, Antònia Llairó, Emma Prims, Josep Ferrer, Laia Sastre, Vivette Glover, Nathalie Nanzer

**Affiliations:** 1 Faculty of Psychology, Education and Sports Sciences Blanquerna, Research Group of Couple and Family (GRPF), Universitat Ramon Llull, Barcelona, Spain; 2 Bruc Salut, Barcelona, Spain; 3 Department of Roquetes-Canteres Primary Care Center, Catalan Public Health, Barcelona, Spain; 4 Institute of Reproductive and Developmental Biology, Imperial College London, London, United Kingdom; 5 Child and Adolescent Psychiatry Service, Geneva University Hospitals, Geneva, Switzerland

**Keywords:** disadvantaged population, Ðother-infant bond, parenthood-centred psychotherapy (PCP), perinatal depressionÚnd anxiety (PNDA), perinatal mental health

## Abstract

**Introduction:**

Untreated depressive-anxiety symptoms during pregnancy can lead to recurrent or chronic maternal depression, with negative consequences for child development and the mother-infant bond. The hypothesis is that Parenthood-Centred Psychotherapy reduces depressive-anxiety symptoms and promotes the mother-infant bond in a disadvantaged population, with high rates of these symptoms. Methods. This study was conducted between 2015 and 2023 in a Primary Health Care Centre in Barcelona in 160 women with prepartum depressive-anxiety symptoms, focusing on outcomes for the mother, the child and the parent–infant bond. At-risk pregnant women (Edinburgh Postnatal Depression Scale ≥ 9 and/or The State–Trait Anxiety Inventory ≥ 40) were assigned by recruitment order to either a treatment group receiving six individual psychotherapy sessions (three during pregnancy and three in the first six postpartum months) or a control group receiving standard perinatal primary care. Maternal depression and anxiety were assessed at baseline (pregnancy), and when the child was 2, 6, and 18 months old. All assessments were conducted by independent blind raters at the final 18-month post-partum, along with evaluation of the Parent–Infant Relationship using the Global Assessment Scale and child development through the Ages and Stages Questionnaire. A two-sample t-test was applied to compare outcomes at different time-point assessments, and Chi-square test to study risk factors between the treatment and control groups, changes in high risk and moderate risk at depressive symptoms and at baby development.

**Results:**

These support the hypothesis that the treatment group showed a significant reduction in depressive symptoms (*p* = 0.011) and state anxiety (*p* = 0.015), compared with the control group, with between-group differences increasing over the follow-up period (*p* < 0.001). At 18 months, infants in the treatment group scored better on three of the five ASQ-3 developmental domains. Significantly, higher quality relationship scores were obtained in the treatment group (*p* < 0.001).

**Discussion:**

Parenthood-Centred Psychotherapy achieves significant improvements not only in maternal depressive-anxiety symptoms but also in infant developmental outcomes and the quality of the mother-infant relationship. These findings suggest this psychotherapy may act as a developmental protective factor and foster a secure and responsive relational environment in early life.

## Introduction

Perinatal depression and anxiety (PNDA) is a worldwide public health issue affecting around 15–20% of pregnant women (WHO, 2022; Barat et al., 2024; Liu et al., 2022).

Untreated anxiety-depression can have negative impacts on child neurodevelopment and the mother-infant bond in pregnancy and the postnatal period (Chen et al., 2024; Goodman, 2020; Lautarescu et al., 2020; Pilowsky et al., 2008; Tuovinen et al., 2018; Van Niel and Payne, 2020; Tusa et al., 2025; Gentile, 2017).

The prevalence of perinatal depression is approximately twice as high in low-and middle-income countries compared to high-income ones (Gelaye et al., 2016; Gomà et al., 2021; Wang et al., 2021), highlighting the importance of implementing perinatal therapeutic interventions in under-resourced populations.

Pioneering research by Leuzinger-Bohleber (2021, 2024) was influential in developing knowledge and comprehension in migrant populations and demonstrating that the long-term benefits of depression treatment are more closely associated with psychoanalytic interventions than standard cognitive behavioural therapy. Scientific literature emphasises the need for high-quality research in perinatal interventions (Matvienko-Sikar et al., 2023; Pettman et al., 2023). Adina et al. (2022) highlighted the lack of research on the impact of perinatal interventions that simultaneously target the mother, the child, and the parent–child bond. Sleed et al. (2023) also mentioned in a separate metanalysis that psychodynamic and psychoanalytic interventions are effective in improving outcomes for very young children and their caregivers. Specific intervention techniques are needed such as Parenthood Centred Psychotherapy (PCP) that addresses the roots of depression and anxiety from the prenatal period and builds on mother-infant psychotherapies developed by Cramer and Palacio-Espasa (1993). Two previous pilot trials conducted in Geneva (Moayedoddin et al., 2013; Nanzer et al., 2012a,) suggest that PCP is an effective therapy for treating perinatal depression and anxiety.

The present work was carried out to expand on the Geneva School studies by replicating it in Primary Health Care in Barcelona (Spain) with a larger sample including a control group (CG) and an assessment of child development. Another novelty is the application of the PCP in a disadvantaged population with high rates of depressive-anxiety symptomatology, migration and accumulated risk factors (Gomà et al., 2021). The therapeutic strategy was based on active involvement and psychodynamic support with the assistance of an interdisciplinary public health care team. The importance of screening for antenatal depression and initiating timely, pregnancy-based interventions to reduce risks for both mother and infant was highlighted by Luciano et al. (2022).

Following the recommendations of these recent metaanalyses, the present study was designed to focus directly on the depressive-anxiety symptoms from pregnancy, targeting the mother-infant dyad across three dimensions: mother’s symptomatology, infant development and the mother-infant relationship.

Our hypothesis was that PCP would significantly improve mothers’ depressive-anxiety symptoms, the mother-infant relationship and child development with respect to those in the CG. PCP is expected to be of great benefit to pregnant women and new mothers and their babies as it focuses on maternal representations and mother-infant interactions, particularly in a vulnerable population during the transition to parenthood.

## Materials and methods

### Sample

Recruitment took place at the Roquetes-Canteres Primary Care Centre (PCC) in a low-income neighbourhood in Barcelona (1.46 multiple deprivation index) which covers a population of 18,724 people, with 45% of the population of child-bearing age. Family income is below half of the Barcelona average (Barcelona 50.4%), unemployment rate is higher than average, and 31% of inhabitants are immigrants (Muñoz and Barbieri, 2016).

Recruitment was carried out between September 2015 and October 2023, interrupted by the COVID-19 pandemic.

All pregnant women with PNDA symptomatology consulting Roquetes PCC were invited to participate in the study. After an introductory visit to explain the study and inform participants of their right to withdraw from it at any time, an ad-hoc Socio-demographic and Risk Factors Data Questionnaire for maternity (SRFDQ) (Gomà et al., 2021) was administered through an interview. Subsequently, each woman was screened for depression (Edinburgh Postnatal Depression Scale, EPDS, Cox et al., 1996) and anxiety (The State–Trait Anxiety Inventory, STAI, Spielberger et al., 1970). Where PNDA results were positive, the participant was included in the study by an independent administrator by simple 1:1 allocation to the therapeutic group (TG) receiving brief PCP or the control group (CG). The CG received standard reproductive care, consisting of consultations with a public health team including a general psychologist and a midwife in a separate specialised centre. All participants provided signed informed consent.

Inclusion criteria:

- EPDS score ≥9 and/ or STAI-trait and state score >39
- From 13 weeks gestation
- Aged 18 years or over

Exclusion criteria:

- Inability to communicate in Catalan, Spanish, French or English
- Intellectual disability or psychiatric pathology severe enough to require intensive treatment

**Figure 1:**
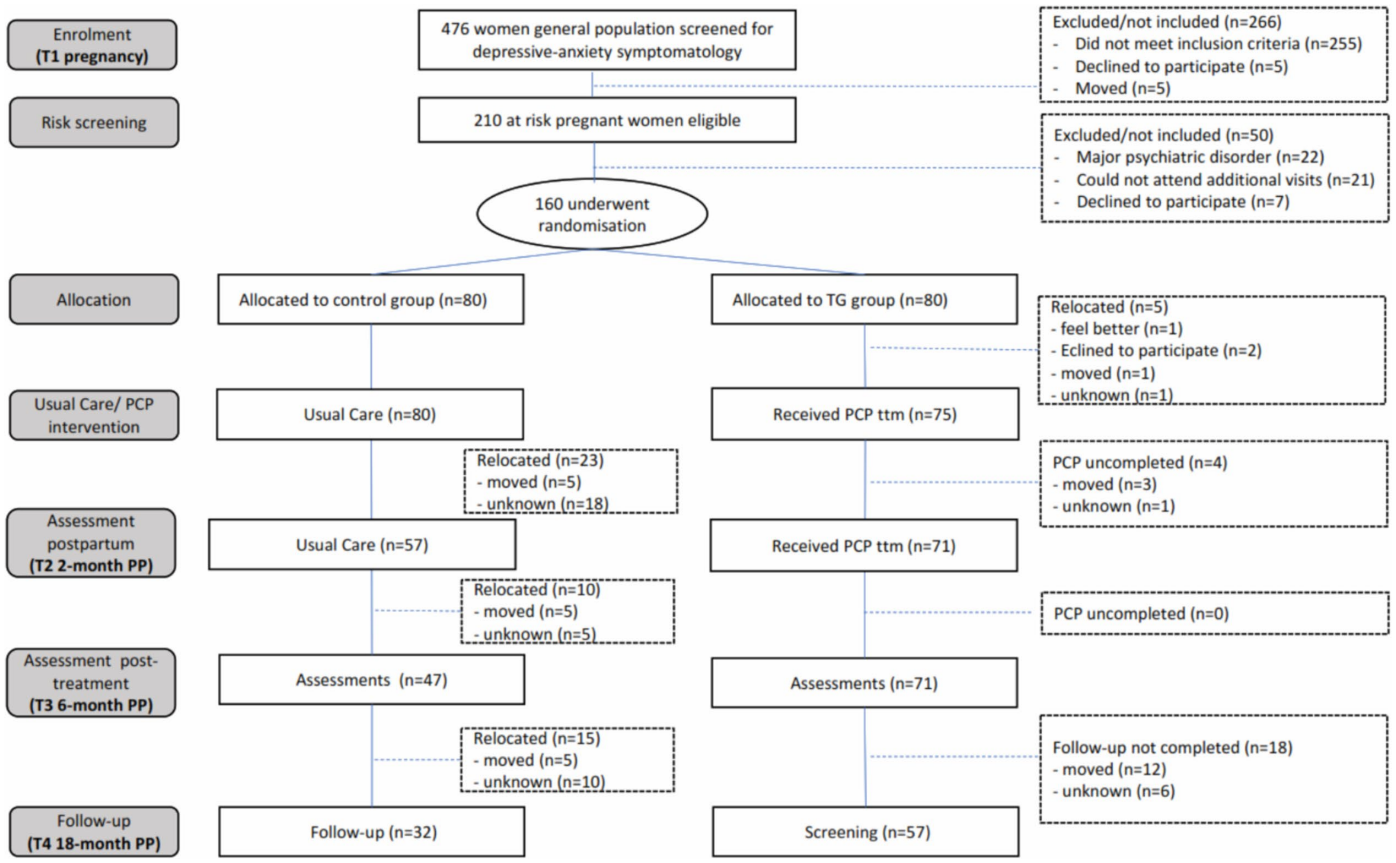
Consort diagram. PP, Postpartum.

Of the 476 pregnant women attended by midwives in the PCC, 210 screened positive for depressive-anxiety symptomatology. A total of 160 met the above criteria and agreed to take part in the study. Consort diagram (Figure 1) shows the flow chart of participants. A total of 160 women attending an appointment with a midwife were alternately allocated into two groups by an independent administrator. Five women declined to participate prior to the intervention, leaving the TG with a total of 75 women.

### Design and assessment

A quasi-randomised longitudinal controlled trial was conducted with pregnant women, screened for depression and anxiety symptoms. Pregnant women attending their first appointment with the midwife were informed about the study and screened for depression and anxiety. At-risk women were randomised by an independent administrator to avoid selection bias: The therapeutic group (TG) received a brief PCP intervention, while the control group (CG) received standard reproductive care as detailed above. General care was provided to all pregnant women by a multidisciplinary team (GP, nurse, midwife, paediatrician, physiotherapist, social worker) created at Roquetes PCC as part of the research design, following WHO guidelines (WHO, 2022).

### Parenthood-Centred Psychotherapy (PCP) study design

PCP is a brief psychoanalytic intervention designed for research in psychoanalysis using techniques described by Cramer and Palacio-Espasa and manualised by the Geneva School, detailing the methods, instruments and procedures (Nanzer et al., 2012a).

In this PCP study, 4 therapists were engaged as experienced psychoanalytic psychotherapists trained in applying the PCP protocol. The psychoanalytic team has weekly supervision sessions with a training psychoanalyst during which the progress of each case is reviewed and adherence to the treatment protocol is ensured.

The TG participated in six psychotherapy sessions. The first 3 sessions take place during the second and third trimester of the pregnancy, followed by 3 mother-infant sessions during the first 6 months postpartum.

The psychotherapist, through analytic listening, identifies the main focus of maternal internal conflict, accepting the transference without interpretation. This is used to understand object relations in the pregnant woman, while countertransference is an essential element in comprehending the patient’s inner world. The mothers’ representations, dreams and projections are analysed to identify the unconscious beliefs related to parenthood, as well as the elaboration of internal conflicts that are at the heart of PCP. The baby is present at all times, during pregnancy in the mother’s mind and womb, and later in the postpartum sessions.

### Description of the PCP intervention technique

The therapist is active in creating a therapeutic alliance, defining the pregnant woman’s predominant anxiety defences to address the underlying trauma and fears associated with motherhood and allow recognition and elaboration of the troubling maternal representations of herself and her future baby. These aspects are often related to the woman’s early-life experiences and her relationship with her own mother.

The internal conflicts generally observed during motherhood revolve around four main themes: the fear of not being a good mother, which manifests as self-denigration; fear of being judged, which is regularly accompanied by a strong sense of guilt; the fear of repeating painful childhood experiences, together with the reactivation of representations and affects linked to one’s own early relationships; and excessive expectations and unrealistic parental ideals, leading to shame and self-reproach. Such internal conflicts are connected to unconscious or insufficiently elaborated entrenched grievances. These may involve unresolved losses, such as the death of a parent or the loss of an invested relationship, but also disillusionments concerning the image the parent had formed of their own parents, of themselves, or of their child.

The intervention leads to an improvement in the mother’s capacity for reflection and insight by alleviating anxiety and guilt, allowing her to become active in her own therapy and to free mental space for the child.

During the first session, the therapist explores the themes and representations concerning parenthood with the mother. The woman is free to associate while the therapist listens in an analytic, nondirective way. The aim is to determine the central element in the psychic conflicts. During the sessions before delivery, the therapist is more active and helps the woman to elaborate her conflicting representations and deepen her understanding of past experiences (Pellet and Nanzer, 2014; Llairó et al., 2023).

Sessions 4 to 6 occur after the baby arrives. In session 4, new concerns appear and the mother is able talk about them. In the following sessions, the antenatal internal conflicts are frequently externalised, acted out in the mother–infant relationship and observed by the therapist. The therapist relates maternal representations in pregnancy to the interactions between the mother and her infant. The mother is guided towards identifying the meaning underlying the interactions. The last two sessions permit the mother to better elaborate her feelings as a mother and build the confidence that will sustain them as they separate from the therapist and the infant becomes more autonomous (Table 1).

**Table 1:** Description of each session.

1st session: initial evaluation and early treatment	Exploration of the most usual themes/representations concerning parenthood and its identifications; the future mother is free to associate while the therapist listens in an analytic, non-directive way.
2nd session: establishing the focus of conflicts	The therapist works on identifying the main focus of maternal themes/representations (maternal ideals, real or fantasised losses and bereavements) and actively confronting the future mother with her unbalanced or distorted representations.
3rd session: working through anxieties around the birth	Previous to the partum: treating partum anxieties and reinforcing the continuity of PCP in the postpartum process
4th session: elaboration of conflictive mother-infant interactions	Mother–infant interactions can be observed and discussed. The mother gains insight about her internal conflicts; the therapist helps the mother to recognise the current repetition of past schemes; both therapist and mother are active.
5th session: re-elaboration	Working through the new concerns and reflections. The mother is guided to identify the meaning underlying the interactions with her baby, and to better elaborate her feelings as a mother.
6th session: conclusion process	Working through separation, which permits the mother to better elaborate her feelings as a mother and the process of separation from the therapist

The Geneva School published a manual detailing the methods, instruments and procedures (Nanzer et al., 2012a).

### Measures

**Figure 2:**
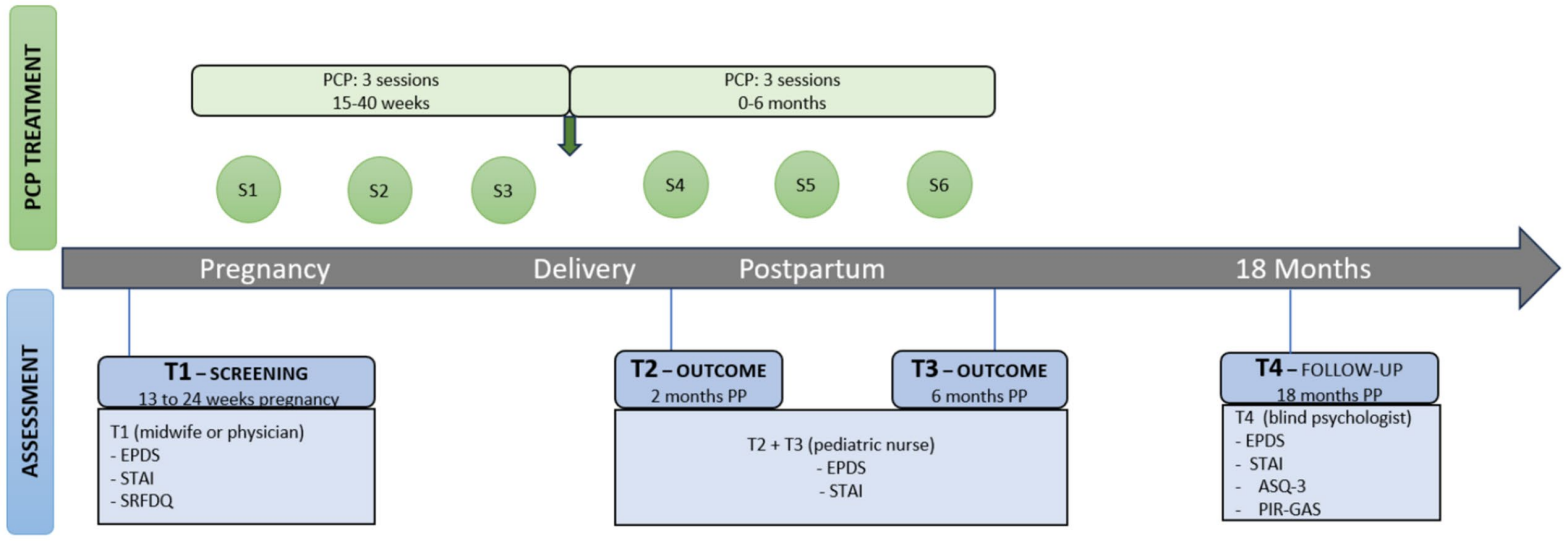
Timeline, assessment, and PCP intervention. ASQ-3, Ages and Stages Questionnaire; EPDS, Edinburgh Postnatal Depression Scale; PCP, Parenthood-Centred Psychotherapy; PP, Postpartum; SRFDQ, Sociodemographic and Risk Factors Data Questionnaire; STAI, State–Trait Anxiety Instrument.

Baseline assessment screening (T1) was conducted by the midwife or, occasionally, the GP. Outcomes were then measured at 2 (T2) and 6 months (T3), and at 18-month postpartum follow-up (T4). PCP ended at the 6-month assessment. The measurements (T1, T2, and T3) were performed by health professionals (midwives and paediatric nurses) whereas the 18-month postpartum measures (T4) were carried out by independent blinded psychologists (Figure 2).

Blind follow-up when the infant is 18-months old allows assessment of the child’s development, the mother-infant bond and the changes in the mother’s depressive-anxiety symptoms.

### Study instruments

*Sociodemographic and risk factors data questionnaire (SRFDQ)* is an interview created ad-hoc by the research team and administered during the first midwife pregnancy interview. It collects data on the following variables: socio-demographic (housing, nationality, age, educational level, employment status), parenting indicators (terminations, planned or unplanned pregnancy, and number of children), psychological data (partner support, family support), mental health history (previous mental health care, psychotropic medication, substance use, mistreatment in infancy, violence and abuse) and important family losses (Gomà et al., 2021).

*The Edinburgh Postnatal Depression Scale (EPDS)* (Cox et al., 1996) was initially validated to assess depressive symptomatology in the postnatal period. It was later validated as a reliable means of identifying symptoms of depression in pregnant women. During pregnancy, a score equal to or greater than 9 is considered to indicate risk. The threshold of 13 was used to discriminate high risk (HR > =13) and moderate risk (MR < 13), as described in the version validated in a Spanish population (Ascaso et al., 2003).

*The State–Trait Anxiety Inventory (STAI)* (Spielberger et al., 1970) is one of the instruments used most frequently to measure anxiety. It differentiates between situations causing temporary anxiety, and permanent anxiety as a trait. It has been shown to be a valid instrument for the measurement of anxiety in different populations and has been validated in a Spanish adult population (Guillen-Riquelme and Buela-Casal, 2014). A score equal to or greater than 39 is used as a cut-off point.

*The Ages & Stages Questionnaire (ASQ-3 Spanish version)* covers children aged 1 to 66 months with 21 age-specific questionnaires (Pomés et al., 2016). Each includes six scored items in five developmental areas: Communication, Gross motor, Fine -motor, Problem-solving and Personal-social (Squires and Bricker, 2009), with scores ranging from 0 to 60 and defined cut-off points for each age. At 18-months old, means of healthy development were used as a threshold defining high risk (HR) and moderate risk (MR). Widely used in paediatrics and primary care, the ASQ-3 is supported by evidence for its efficacy (Sheldrick et al., 2020).

*The Parent–Infant Relationship Global Assessment Scale (PIR-GAS)* The Parent–Infant Relationship Global Assessment Scale (PIR-GAS) is a 100-point instrument providing a continuous scale of the quality of adaptation in the parent-infant relationship, ranging from “dangerously impaired” to “well-adapted.” The PIR-GAS is a supplement to the DC-03 diagnostic classification (Zero to Three, 2005).

### Data analysis

The SPSS statistics package for Windows V20 (IBM Corp, 2011) was used to carry out the statistical analyses. We used an alpha level of 0.05 for all statistical tests.

Chi-square was applied to describe differences in risk factors between TG and CG and compare them in dichotomic variables and two-sample t-test when continuous (age, number of children and accumulated risk factors).

An ANOVA, comparing the TG and the CG at four scheduled time-points in terms of mothers’ depression-anxiety symptomatology. Comparison of means for independent samples (two-sample t-test) was applied to study child development and quality of parent-infant relationship at 18 months old (T4 follow -up) comparing both the TG and CG groups.

Depression symptomatology was divided into two groups HR and MR (Ascaso et al., 2003), Chi-square was applied to measure changes in both mother groups as well as HR and MR in baby development at 18 month old follow-up. Pearson correlation was calculated to study the difference on EPDS (from pregnancy (T1) until 18 m (T4)) and the quality of the relationship (PIR-GAS).

## Results

### Participant characteristics: TG and CG

The TG and the CG groups were compared regarding general sociodemographic aspects and determinant risk factors for depression and anxiety (Gomà et al., 2021). While the groups are similar in terms of cumulative risk factors and most items, they differ in terms of unemployment and mental health history. A complete list of differences can be seen in Table 2.

**Table 2:** Sociodemographic characteristics and TG and CG risk factors.

Sociodemographic characteristics	TG		CG		Mean comparison
	Mean	SD	Mean	SD	*p* value
Age (mother)	29.28	5.8	29.33	5.2	0.954
N° children	0.70	0.74	0.89	0.98	0.173
Accumulated risk factors	5.71	2.2	5.21	3.2	0.250

Risk factors	N	%	N	%	*p*
Living in one room	11	13.8	15	18.8	0.521
Migration	45	56.2	43	53.8	0.874
No basic education	17	21.2	16	20.0	1.000
Unemployment	20	25.0	34	42.5	0.029**
Previous miscarriage	37	46.2	36	45.0	1.000
Voluntary pregnancy termination	24	30.4	19	23.8	0.376
Lack of partner support	16	20.0	10	12.5	0.284
Lack of family support	14	18.2	11	13.8	0.516
Lack of social support	5	6.2	7	8.8	0.756
History of mental health problems	30	37.5	16	20.0	0.023**
Psychotropic medication	21	28.4	15	18.8	0.185
Previous experience of violence	21	26.2	13	16.2	0.176
Mistreatment in infancy	21	26.2	11	13.8	0.074
Stressful events in the last 24 months	44	55.0	40	50.0	0.635
Living alone	2	2.5	1	1.2	1.000
Miscarriage	19	24.1	20	25.0	1.000
Unplanned pregnancy	32	40.0	35	43.8	0.749
Does not attend accompanied	51	65.4	44	62.0	0.734
History of physical health	7	8.8	5	6.2	0.756
OH consumption	1	1.2	0	0.0	1.000
Tobacco consumption	6	7.5	12	15.0	0.210
Drug consumption	0	0.0	1	1.2	1.000
Loss in the last 2 years	32	40.5	30	37.5	0.746
Serious illness in past 2 years	28	35.0	17	21.2	0.078
More than 4 risk factors	61	76.2	53	66.2	0.221

**p < 0.005.

Outcomes show the pattern of depressive-anxiety symptoms at each time point for the TG and CG mothers, and development in the TG and CG infants.

### Mothers’ PNDA symptomatology

Mothers who received PCP exhibited improvements in depressive-anxiety symptoms after childbirth (T2), reaching subclinical levels, while the control group maintained elevated scores. Between-group differences increased at the end of treatment.

**Figure 3:**
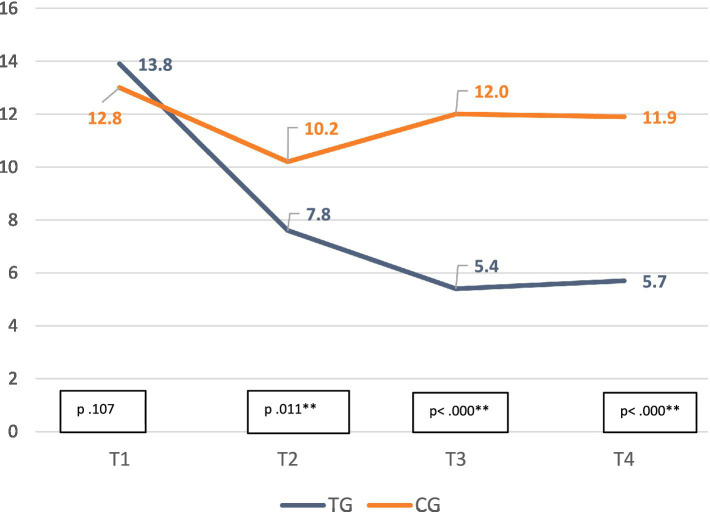
Change in depression score (EPDS) means from pregnancy to 18-months. T1: Pregnancy; T2: 2-month postpartum; T3: 6-month postpartum; T4: 18-month postpartum.

In terms of depression, comparing the TG and the CG EPDS scores by applying an ANOVA, significant differences are noted between T1 and T2 but also after conclusion of PCP, between T2 and T3 (T2 *p* = 0.011). In the TG, improvement was observed at 6 months (T3 *p* < 0.000) and maintained at T4 follow-up (*p* < 0.000) when comparing TG and CG means. See Figure 3.

**Figure 4:**
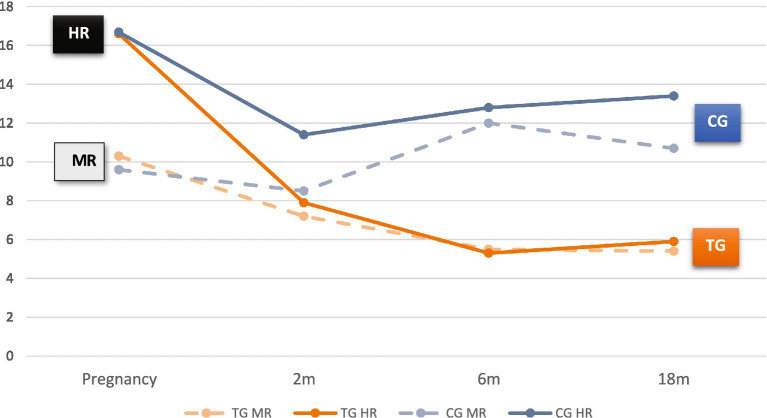
Changes in mothers’ depression scores (EPDS) from pregnancy to 18 months: HR and MR group comparison. EPDS: Edinburgh Postpartum Depression Scale; MR (EPDS 9 to 12), HR (EPDS = > 13).

*Depressive high-risk scores*: Figure 4 shows two subgroups according to depressive scores at T1: Moderate Risk (MR) when EPDS<13 and High Risk (HR) when EPDS = > 13. The mean score of the HR subgroup decreased to that of the MR subgroup in the TG from T2 onwards, whereas in the CG a distinct trend developed, maintaining a high mean EPDS at T3 and T4.

**Figure 5:**
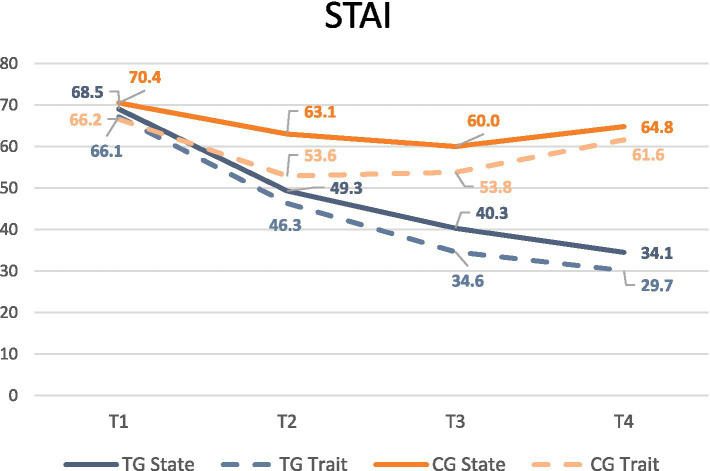
Anxiety scores means from pregnancy to 18-month. T1: pregnancy; T2: 2-month postpartum; T3: 6-month postpartum; T4: 18-month postpartum.

Anxiety symptoms (Figure 5): The same pattern was found for State Anxiety symptomatology with a significant reduction from 2-month postpartum (T2 p 0.015; T3 *p* < 0.000; T4 *p* < 0.000). In Trait Anxiety, the differences appeared clearly at 6-month postpartum on completion of treatment (T3 *p* = 0.001). At follow-up, Trait Anxiety in the TG has diminished to less than half, although in the CG it remained similar to that in pregnancy (T4 p < 0.000). At 18-month follow up (T4), anxiety scores continue to slightly decrease in the TG while increasing in CG (Table 3).

**Table 3:** Anxiety scores from pregnancy to 18-month: group comparison.

Assessment time point		STATE			TRAIT		
		TG	CG	*p* value	TG	CG	*p value*
Pregnancy	T1	68.5	70.4	0.659	66.1	66.2	0.988
2 m PP	T2	49.3	63.1	0.015**	46.3	53.6	0.207
6 m PP	T3	40.3	60.0	< 0.000**	34.6	53.8	0.001**
18 m PP	T4	34.1	64.8	< 0.000**	29.7	61.6	< 0.000**

T1: pregnancy; T2: 2-month postpartum; T3: 6-month postpartum; T4: 18-month postpartum. ** Significance.

### Infant development and mother-infant bond

*Infant development*: At 18-months (T4), significant differences were observed in ASQ scores in 3 domains: Communication skills (p < 0.000), Gross motor (*p* = 0.048) and Problem-solving (p < 0.000). Results for Personal-social and Fine-motor development were also better but not statistically significant.

*The parent-infant relationship* (PIR-GAS) was significantly different when comparing TG and CG (p < 0.000) with a significantly higher quality mother-infant relationship in the TG, compared to the mean score in the CG, which was significantly perturbed and showed greater outcome variability (Table 4).

**Table 4:** Comparison in infant development (ASQ) and mother-baby relationship (PIR-GAS) between the TG and CG at 18 months (T4).

ASQ and PIR-GAS	ASQ-3 and PIR-GAS comparison between the TG and the CG		
	TG	GC	*p* value
Communication	46.8 (8.1)	33.8 (12.6)	0.000*
Gross motor	53.2 (8.9)	47.9 (15.4)	0.042*
Fine motor	52.1 (7.0)	49.0 (11.6)	0.109
Problem-solving	47.9 (7.4)	38.7 (14.3)	0.000*
Personal-social	53.9 (7.6)	50.3 (9.8)	0.052
PIR-GAS	85.45 (7.2)	67.7 (12.8)	0.000*

** Significance.

**Figure 6:**
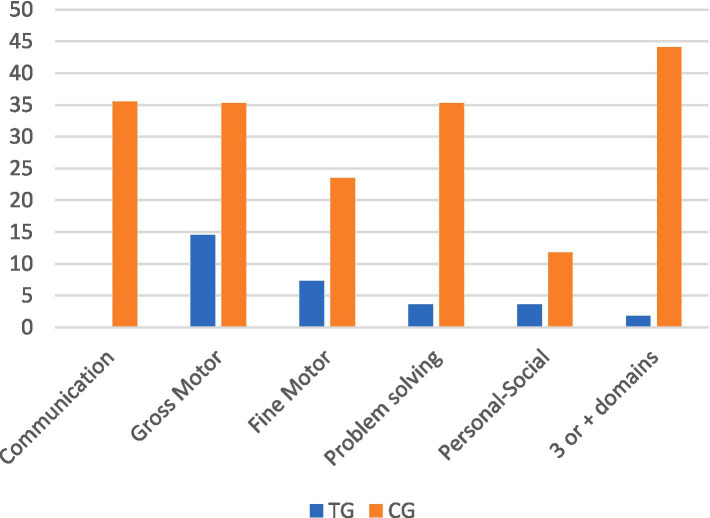
Percentage of at-risk infants comparing TG and CG in each ASQ-3 domain plus accumulated risk (3 or more).

ASQ-3 allows children to be classified into two categories across 5 domains: risk “R” (need to be monitored or below expectations) or no risk “NR.” In the CG, 35% of children were at risk in Communication and Problem-solving whereas in the TG 0% needed to be monitored in Communication and only 3.6% in Problem-solving, with significant differences noted when applying a chi-square test (*p* > 0.000). Mother-infant relationship (PIR-GAS) can also be categorised into risk (= < 80, perturbed) or no risk (81–100). If accumulated risk in 3 or more ASQ-3 domains plus PIR-GAS were detected, we considered infant development as being in the high risk “HR” category. This resulted in only 1 child in the TG (1.8%) compared to 15 children (44.1%) in the CG (*p* > 0.000) being classified as HR, as shown in Table 5 and Figure 6. In terms of higher accumulated risk, 4 domains or more, 8 children (23.5%) from the CG were considered HR with significant differences observed with respect to the TG when applying a chi-square test (p 0.002), as shown in Table 5 and Figure 6.

**Table 5:** Risk and no-risk in ASQ-3 plus PIR-GAS.

ASQ and PIR-GAS		R		NR		Chi-square
		N	%	N	%	
Communication	TG	0	0	55	100	> 0.000**
	CG	12	35.3	22	64.7	
Gross motor	TG	8	15	47	85.5	*0.063*
	CG	12	35.3	22	64.7	
Fine motor	TG	4	7.3	51	93	0.84
	CG	8	23.5	26	76.5	
Problem-solving	TG	2	3.6	53	96	> 0.000**
	CG	12	35.3	22	64.7	
Personal-social	TG	2	3.6	53	96	*0.316*
	CG	4	11.8	30	88,2	
PIR-GAS relationship	TG	1	1.8	54	98.2	> 0.000**
	CG	15	44.1	19	55.9	

** Significance.

As mentioned previously, significant difficulties were found in 3 or more areas of development and/or the mother-baby relationship in 15 children from the CG.

When applying a Pearson correlation between EPDS and PIR-GAS, high statistical significance was found in TG; the lower the mother’s EPDS score, the higher the quality of parent-infant relationship (PIR GAS). No statistical significance was found in CG. When studying (time?) the difference on EPDS (from pregnancy (T1) until 18 m (T4)) and the quality of the relationship (PIR-GAS), in the TG we obtained *r* 0.451 p 0.001 whereas in the CG *r*.092 and p 0.635. In the TG, the greater the drop on the EPDS since pregnancy, the higher the levels in the quality of the relationship were observed 1 year after treatment. This pattern was not seen in the CG (Table 6).

**Table 6:** Pearson correlations between depression symptoms (EPDS) and parent-infant relationship (PIR-GAS) in TG and CG.

	TG		CG	
	*r*	Sign.	*r*	Sign
Pregnancy	0.164	0.226	0.054	0.760
2 m	−0.107	0.435	0.059	0.767
6 m	−0.103	0.450	−0.104	0.604
18 m	−0.478**	> 0.000**	−0.217	0.249
Difference drop (pregnancy-18 m)*	0.451**	0.001**	0.092	0.635

** Significance.

## Discussion

This study continues the work of the Geneva School and demonstrates that PCP outcomes in a disadvantaged population are in line with the findings of the Geneva pilot studies in a more advantaged population, especially in those women at high risk for PNDA. As hypothesised, mothers and infants receiving brief PCP in the TG showed significant improvements during early postpartum (2-month-old infant) that continued until the end of the treatment when the baby was 6 months old. It should be mentioned that when improvements are observed at 6 months, these generally persist until 12 months from treatment completion with very little change. These results are consistent with the findings of two previous pilot studies (Moayedoddin et al., 2013; Nanzer et al., 2012a,b;). Interestingly, the high-risk (HR) TG subgroup showed a substantial reduction in symptomatology. This suggests that women with severe PNDA symptomatology benefit as much, if not more, from PCP. Psychotherapy research also suggests that individuals with higher baseline symptom severity may show greater neurobiological modulation during treatment. Joensuu et al. (2016) found that changes in serotonin transporter regulation were more pronounced in patients with more severe initial symptoms, indicating a state-dependent compensatory mechanism. This supports our observation that high-risk mothers benefited markedly from PCP during pregnancy.

In the follow-up at 18-months postpartum, mothers’ PNDA symptomatology maintained their T3 EPDS scores, indicating sustained benefits of PCP after its conclusion in the TG and the absence of spontaneous improvement in the CG. The persistence of medium-to-long-term improvement is a common feature of psychoanalytic psychotherapies, as shown by Leuzinger-Bohleber (2024).

The associated risk of long-term PNDA is well-documented (Biaggi et al., 2016), as are the epigenetic consequences (Glover, 2023) for the mother-infant bond (Moayedoddin et al., 2013; Della Vedova et al., 2023). It is well known that perinatal depression and mother-infant relationship disorders constitute major risk factors for developmental disorders of childhood (Lautarescu et al., 2020; Osborne et al., 2018; Pilowsky et al., 2008; Van Niel and Payne, 2020), as well as psychiatric disorders in children, adolescents and adults (Chen et al., 2024; Goodman, 2020; Hughes et al., 2017). These effects may also manifest across generations.

Mother-infant bond is a strong predictor of children’s mental health (Keskinen et al., 2018). At 18 months, higher quality of parent-infant relationship scores were obtained in the TG than in the CG. Those PCP families with a greater decrease in depression scores from pregnancy experienced higher quality mother-infant relationships. The healthy developmental trajectory found in children in the TG indicates that benefits may result from commencing psychotherapies in pregnancy for at-risk mothers. Introducing and expanding this approach in public health would potentially help to prevent children developing a range of disorders and reduce family suffering. A recent meta-analysis indicated that psychoanalytic interventions at this stage improve the developmental course of the child and their caregivers (Sleed et al., 2023).

Providing PCP from pregnancy to 6 months postpartum can prevent the cumulative effects of depressive-anxiety symptoms and promote positive child neurodevelopment at 18 months. This may support healthy developmental trajectories and mitigate the poorer child neurodevelopment reported in some studies (Tuovinen et al., 2018; Chen et al., 2024). It is important such children are referred to suitable programmes in early-life care.

This study focuses on the first mother-infant bond during the transition to parenthood. The PCP approach is both brief and effective, as evidenced by the decrease in depressive-anxiety symptoms in the mother and the low drop-out rate during the intervention. Initiating treatment during pregnancy, when psychic transparency enhances high psychic mobility, allows for considerable improvements. These findings underscore the importance of starting PCP prenatally, consistent with previous results reported by Nanzer et al. (2012a) and Adina et al. (2022). As mentioned in the introduction, this work builds on the research and pioneering psychotherapies of Cramer and Palacio-Espasa. It is important to note that this study, following WHO 2022 guidelines, was carried out in Primary Health Care (PHC) within an interdisciplinary network (GPs, paediatricians, midwives and social workers), together with psychoanalytic psychotherapists, providing support to mothers and families in their community. Interdisciplinary interventions are essential in providing comprehensive care for pregnant women, new mothers and their families. Together with the Geneva School studies, this study, carried out in a vulnerable population, demonstrates the potential for the intervention to be effective in distinct areas and contexts.

The strengths of PCP are that the therapy focuses on the emotional challenges of transitioning to motherhood while promoting healthy baby neurodevelopment and the mother-infant bond. Due to its psychodynamic orientation, this approach allows for individualised care, considering the specific needs of each dyad. The novel implementation in a vulnerable sample within an interdisciplinary framework shows the potential benefits to other disadvantaged populations. Public health policies should encourage brief perinatal psychotherapies as a standard component in health care.

### Limitations

The implementation of PCP requires the availability of psychotherapists who are specifically trained and supervised, which could limit its general dissemination.

## Conclusion

This psychoanalytic study addresses urgent social issues with its aim of decreasing perinatal depressive-anxiety symptoms, ensuring that the child meets developmental milestones at 18 months old.

These results emphasise the need for maternal mental health care in pregnancy as a public health priority, as suggested by Chen et al. (2024). Further research using larger samples and longer follow-up are needed to corroborate the clinical benefits of prenatal interventions. Policy-makers need to be made aware of the need for preventive measures and investment in treatment in this area to improve mothers’ health and infant development.

- Detection and treatment of depressive-anxiety symptoms in pregnancy is needed in all pregnant women, especially in disadvantaged populations.
- This paper confirms that PCP is a suitable framework for perinatal interventions through an interdisciplinary network in Primary Health Care.

Based on these findings, some recommendations can be made for treating perinatal anxiety and depressive disorders, preventing parent-infant relationship difficulties, and reducing the risk of psychopathology in children:

- Intervene as early as possible, during pregnancy, to prevent difficulties arising later.
- Apply specific perinatal interventions administered by trained psychotherapists.
- Select interventions that address both prenatal parental psychopathology and the parent-infant bond; here PCP has been shown to be highly effective.
- Include women with high depression scores in brief psychotherapies, as they respond to the intervention as well as those with moderate scores.
- Implement the intervention using interdisciplinary networks in disadvantaged populations with multiple risk factors, as they are as receptive to PCP as other groups.

## Data Availability

The original contributions presented in the study are included in the article/supplementary material, further inquiries can be directed to the corresponding author.
